# Barriers and facilitators of oral health care utilization among pregnant women: a mixed-methods systematic review

**DOI:** 10.1186/s13643-026-03151-8

**Published:** 2026-04-20

**Authors:** Lekshmi Subramaniam, Parvathy Balachandran, Chandrashekar Janakiram

**Affiliations:** 1 Department of Public Health Dentistry, Amrita School of Dentistry, Amrita Vishwa Vidyapeetham, Kerala, India; 2 JBI Amrita Centre for Evidence Synthesis and Implementation, Kochi, India

**Keywords:** Pregnancy, Oral health, Oral care utilization, Barriers, Facilitators, Mixed method

## Abstract

**Introduction:**

Oral health is an important yet often neglected aspect of antenatal care. Despite evidence linking poor maternal oral health with adverse pregnancy outcomes, pregnant women underutilize dental services due to individual, cultural, and systemic barriers. This is the first systematic review using mixed-methods approach that synthesized quantitative and qualitative evidence on this topic.

**Methods:**

A mixed-methods systematic review was conducted using Joanna Briggs Institute (JBI) methodology and reported per PRISMA guidelines. Eligible studies included those exploring barriers and facilitators to dental service utilization among pregnant women. Searches across six databases and grey literature sources were conducted. Quantitative data were qualitized and integrated with qualitative findings using JBI’s convergent integrated approach. Findings were reported based on Andersen’s Behavioral Model of Health Services Use.

**Findings:**

The review included 55 studies (37 quantitative, 17 qualitative, 1 mixed-methods) from 24 countries. Eight key themes based on Andersen’s Behavioral model emerged. Barriers included misconceptions, safety concerns, dental treatment fear, high costs, lack of insurance coverage, poor referral systems, limited provider training, competing priorities, and cultural taboos. Facilitators included higher education, employment, urban residence, and provider support.

**Discussion:**

This is the first systematic review offering a comprehensive synthesis of the multifaceted barriers and facilitators shaping pregnant women’s decisions to seek oral care. It integrated global prevalence data from quantitative studies with the lived experiences of pregnant women through qualitative studies. Andersen’s model enabled structured interpretation of individual, contextual, and health-related determinants.

**Recommendations:**

It is important to develop culturally sensitive and integrated strategies to enhance maternal oral health service utilization across diverse settings. Policies should mandate integration of oral health into antenatal care, expand dental insurance for pregnant women, and enhance interprofessional training. Further research should explore longitudinal patterns, develop validated tools, and address intersectional disparities in access.

**Systematic review registration:**

PROSPERO CRD42025642722.

**Supplementary Information:**

The online version contains supplementary material available at 10.1186/s13643-026-03151-8.

## Introduction

Pregnancy is a period marked by major physiological, hormonal, and behavioral changes that increases a woman’s susceptibility to health issues including oral diseases [1]. These include gingivitis, periodontitis, and dental caries which can negatively impact the quality of life of pregnant women by disrupting their sleep, diet, and daily activities [2]. Gingival inflammation is found to be present in 36–100% of pregnant women [3]. Poor maternal oral health is linked to serious pregnancy outcomes like preterm delivery, low birth-weight [4, 5], intrauterine growth restrictions [6], and preeclampsia [7]. Additionally, periodontal disease is associated with gestational diabetes which can further aggravate complications like neonatal hypoglycemia, fetal macrosomia, and shoulder dystocia [8]. Maternal cariogenic bacteria are also found to be vertically transmitted to the infant, jeopardizing the child’s oral health [9].

Despite its importance, maternal oral health continues to be a neglected component of prenatal care in many health systems. This is concerning as untreated dental conditions can harm both maternal and fetal health. A key contributor to this is the underutilization of dental services by pregnant women due to lack of awareness, socioeconomic barriers, safety concerns, or “health care system related-reasons” [10]. Pregnancy-related discomfort such as nausea, vomiting, and fatigue, as well as cultural taboos, financial constraints, and dental anxiety discourage pregnant women from seeking oral care [11]. In some cultures, women are expected to endure pain, including the dental pain [12]. Even the absence of female dentist in the locality may create a cultural barrier to seeking oral care [11].

Despite the existence of clinical guidelines for oral care during pregnancy [13], reluctance of dental practitioners to treat pregnant women due to lack of sufficient training, time constraints, hesitancy due to medicolegal risks, and lack of institutional support is a notable deterrent [11]. Additionally, system-level factors like lack of interprofessional collaboration between dental and prenatal care providers, inadequate oral care facilities, and absence of oral care coverage in insurance further limits the use of dental services [14, 15]. Collectively, there is a complex interplay of factors ranging from individual-level barriers to system-level reasons that contribute to inadequate oral care utilization among pregnant women.

Despite these existing challenges, there are several factors that facilitate dental service utilization in pregnancy period. While awareness about the importance of oral health and positive attitude to dental treatment improves oral care seeking at the individual level, effective referral mechanisms and free oral care act as system level facilitators [3, 10, 11, 16]. Another facilitator reported was the insurance coverage for dental treatments [3, 16].

Since there is a myriad of factors that act as barriers or facilitators of oral care utilization in pregnancy, the Andersen’s Behavioral Model of Health Services Use (BM) will provide a structured theoretical framework to understand these [17]. This model suggests that an individual health-related behavior is formed by their perception of vulnerability to the condition, its potential seriousness, the benefits of preventive measures, and the anticipated difficulties in seeking care [17, 18].

Although several quantitative and qualitative studies have examined determinants of oral health care utilization during pregnancy, the existing evidence remains fragmented. Quantitative studies primarily identify statistical associations between socio-demographic factors and service use [3, 19], whereas qualitative studies explore beliefs and lived experiences in specific contexts [11, 20, 21]. However, there is limited integration of these dimensions to explain how individual perceptions interact with structural and system-level determinants. This lack of cross-level integration restricts a comprehensive understanding of the mechanisms underlying care-seeking behavior during pregnancy.

To date, no systematic review has synthesized both qualitative and quantitative evidence to comprehensively examine the full spectrum of barriers and facilitators affecting pregnant women’s utilization of oral health care services across global contexts. A preliminary search of major databases including PROSPERO, MEDLINE (via PubMed), the Cochrane Database of Systematic Reviews, and JBI Evidence Synthesis revealed no current or ongoing mixed-methods systematic reviews on this topic. Moreover, existing reviews lack a robust theoretical framework to explain the interplay between the various determinants of oral health service utilization. To address these limitations, the present systematic review uses a mixed-method approach guided by Andersen’s Behavioral Model of Health Services Use.

## Methods

### Aim

This systematic review aims to answer the question: What are the barriers and facilitators influencing pregnant women's utilization of oral health care services?

### Design

A mixed-method approach of systematic review was chosen to provide a holistic and theory-driven understanding of the factors influencing dental services utilization in pregnancy. This review was conducted according to the Joanna Briggs Institute (JBI) methodology for Mixed Method Systematic Reviews [22] and adhered to the Preferred Reporting Items for Systematic Reviews and Meta-Analyses (PRISMA) guidelines [23]. The review protocol was prospectively registered in the PROSPERO database (ID: CRD42025642722; Date of registration: 05 March 2025). The literature search was conducted between 8 April 2025 and 17 April 2025. The review was conducted from March 2025 till June 2025.

### Eligibility criteria

This review included articles based on the Participants, Phenomenon of Interest, and Context criteria.

#### Participants

This systematic review included qualitative and quantitative studies that explored barriers and facilitators to oral health care utilization among pregnant women aged 18 years and above. There was no limitation based on year of publication, geographic location, cultural setting, socioeconomic status, educational background, trimester of pregnancy, or gravida status. Postpartum women were included if their experiences during pregnancy were discussed.

Studies involving pregnant women with high-risk conditions like HIV/AIDS, cancer, preeclampsia, gestational or preexisting diabetes, a history of preterm birth, stillbirth, recurrent miscarriages, and placenta previa or other major placental abnormalities were excluded to minimize confounding factors. Studies focusing primarily on health care providers were also excluded. For studies with mixed populations, only data relevant to non-high-risk pregnant women were extracted.

#### Protocol deviation

Although the original protocol specified the inclusion of only participants aged 18 years and above, a few studies involving participants aged 15 years and above were included. This deviation was made to capture culturally relevant data from settings where early pregnancy is more common. These were included to cover the real-world scenario.

#### Phenomena of interest

This review focused on the barriers and facilitators of oral health care utilization among pregnant women. In this context, barriers were defined as factors that limited, inhibited, or discouraged the use of oral health services during pregnancy, while facilitators were factors that supported, encouraged, or enabled such utilization. Barriers included fear or anxiety related to dental treatment, cultural or personal beliefs about oral health care during pregnancy, lack of knowledge or awareness regarding the importance and safety of oral care during pregnancy, and financial or logistical constraints. Facilitators encompassed increased awareness and education, affordability of dental services, accessibility and availability of oral care, and support from health care providers. Studies focusing solely on clinical outcomes, interventions, or treatment effectiveness without addressing utilization factors were excluded.

#### Context

Primary studies conducted in any settings that assessed oral health care utilization during pregnancy were considered for inclusion in this review. These settings included community-based environments or health care institutions such as dental clinics, hospitals, maternity centers, public health programs, or even home-based care settings. The review did not impose any limitations based on geographic location, allowing the inclusion of studies from diverse global regions to capture a comprehensive perspective. Similarly, studies conducted in both urban and rural areas were included to ensure the review reflected variations in accessibility, health care infrastructure, and sociocultural factors that may have influenced oral health care utilization among pregnant women.

#### Types of studies

This review considered quantitative, qualitative, and mixed-method studies that investigated factors influencing oral health care utilization among pregnant women. Quantitative studies included those that assessed determinants of oral health care utilization through validated models or other analytical approaches, often via questionnaire-based surveys. Qualitative studies involved research that explored the perceptions, experiences, and attitudes of pregnant women towards oral health care, utilizing methods such as in-depth interviews or focus group discussions.

Mixed-method studies were considered only if the findings from the quantitative and qualitative components could be clearly disaggregated and extracted for analysis. To ensure comprehensive coverage of relevant evidence, grey literature such as academic dissertations and conference proceedings indexed in ProQuest and Google Scholar were also included, provided they met the eligibility criteria. Editorials, opinion pieces, policy briefs, letters to the editor, and case reports were excluded from the review. There was no restriction on year of publication. No language or date restrictions were applied during the search. Non-English articles identified during screening were documented for transparency but were not included in the final analysis due to language limitations.

### Search strategy

A comprehensive three-step search strategy was used to identify both published and unpublished studies. First, an initial limited search of MEDLINE (PubMed) was conducted to identify relevant articles on the topic. The text words contained in the titles and abstracts of these articles, along with the index terms used to describe them were analyzed to develop a full search strategy for MEDLINE (PubMed). This strategy was then adapted for each included database and information source. The final search strategy for each database was developed through consensus among the authors.

In addition, the reference lists of all included articles and relevant systematic reviews were screened to identify any further eligible studies. The databases searched included MEDLINE (PubMed), Scopus (Elsevier), CINAHL (EBSCO), PsycINFO (Ovid), Dentistry & Oral Sciences Source (EBSCO), and Web of Science Core Collection (Clarivate). Gray literature and sources of unpublished studies were explored through ProQuest Dissertations & Theses Global (ProQuest), OAIster (OCLC), and Google Scholar (Google) (Table 3 Additional file). No language or date restrictions were applied. Non-English articles identified during screening were documented for transparency.

### Study selection

**Fig. 1:**
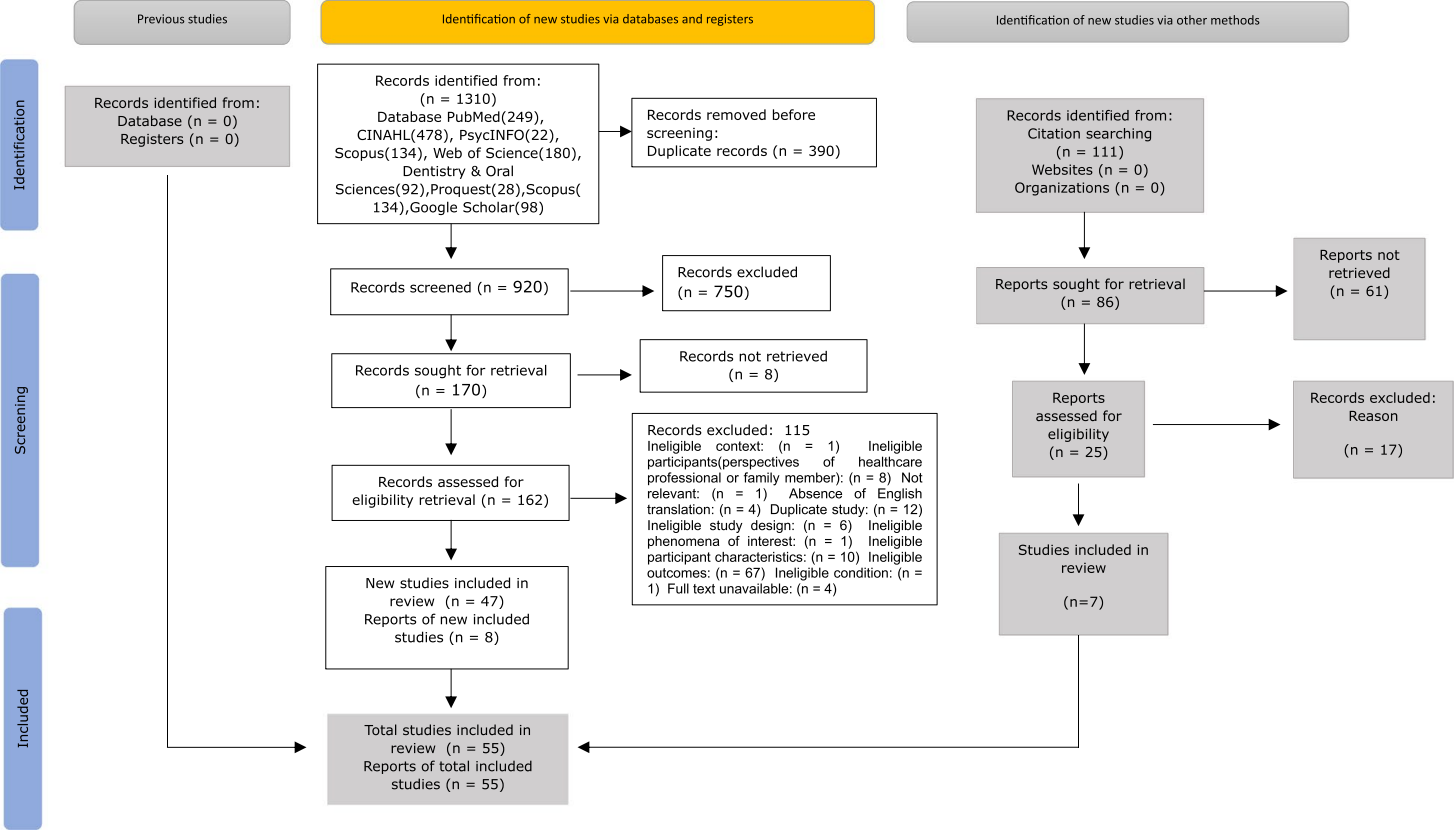
Prisma flow chart

Following the search, all identified citations were imported into Zotero version 7.0.0 [24] and duplicate records were removed. Titles and abstracts were then uploaded into JBI SUMARI, where two reviewers (LS and PB) independently screened them using predefined inclusion criteria. After a pilot screening phase, titles and abstracts were independently reviewed by two reviewers (LS and PB) using predefined inclusion criteria. Full texts of potentially relevant studies were retrieved, and screened by two reviewers (LS and PB) in the JBI System for the Unified Management, Assessment and Review of Information (JBI SUMARI; JBI, Adelaide, Australia) [25]. Reasons for exclusion were documented. Discrepancies between reviewers were resolved through discussion with a third reviewer (CJ). The study selection process and search results were fully documented and presented using a PRISMA flow diagram (Fig. 1).

### Quality appraisal

The quality of each study was independently assessed by two reviewers (LS and PB) using the relevant JBI Critical Appraisal Checklists for Prevalence Studies [26], Analytical Cross-Sectional Studies [27] and Cohort Studies [27], and Qualitative Studies [28]. Prior to formal appraisal, the two reviewers (LS and PB) conducted a pilot calibration exercise on a subset of studies to ensure consistency in applying the appraisal criteria. Any disagreements were resolved through discussion with a third reviewer (CJ). For scoring, each item was assigned a value of 2 if the quality criterion was clearly met, 1 if the information was unclear, and 0 if the criterion was not met. Total quality score was then calculated for each study. Maximum possible scores were 20 for qualitative studies, 22 for cohort studies, 18 for prevalence studies, and 16 for analytical cross-sectional studies. For studies appraised with a checklist, affirmative (“yes”) responses were summed to obtain a total quality score and converted to a percentage of the maximum possible score. Following common practice in previous systematic reviews, scores were categorized as low risk of bias (≥ 75%), moderate risk of bias (50–74%), and high risk of bias (< 50%). For studies assessed using the Newcastle–Ottawa Scale, conventional thresholds were applied (≥ 7 = good quality; 4–6 = fair quality; ≤ 3 = poor quality).

The critical appraisal checklists were used to support structured methodological assessment rather than to impose rigid cut-off thresholds. The total scores were interpreted descriptively as an overall indication of methodological quality, and no studies were excluded based on the numerical score alone. Given the mixed-methods design of this review, different critical appraisal tools were applied according to study design. Because risk-of-bias frameworks differ conceptually and operationally across qualitative and quantitative methodologies, direct comparison or integration of ROB scores across study types was not methodologically appropriate. Therefore, ROB findings were reported descriptively for transparency but were not used to weight findings or conduct stratified analyses.

### Data extraction

Two reviewers (LS and PB) independently extracted data from the included studies using the standardized JBI data extraction tool in JBI SUMARI [29]. It included study population, methods, phenomena of interest, context, and outcomes. Quantitative data comprised statistical outcomes while qualitative data included themes or subthemes accompanied by supporting illustrations and were assigned a level of credibility using the JBI Checklist for Qualitative Research [28]. Any disagreements that arose between the reviewers were resolved through discussion with the third reviewer (CJ).

### Data synthesis and integration

A convergent integrated approach outlined by the Joanna Briggs Institute (JBI) was used to analyze both quantitative and qualitative thematic analysis [22]. The process involved three stages. First, all qualitative findings were extracted and coded for thematic synthesis, while quantitative data were qualitized, meaning they were converted into narrative descriptions to enable integration with qualitative evidence. Next, the qualitized quantitative data and qualitative themes were jointly reviewed (LS, PB, CJ) and organized into categories based on similarities in meaning. Finally, these categories were subjected to thematic synthesis, generating broader integrated findings that reflected evidence from both quantitative and qualitative sources. Throughout this process, the review team (LS, PB, CJ) reread findings for familiarity, refined categories through discussion, and maintained theoretical memos to document decisions and reflections, ensuring transparency and rigor.

#### Theoretical framework: Andersen’s Behavioral Model of Health Services Use

This review is informed by the expanded version of Andersen’s Behavioral Model of Health Services Use (Andersen, 1995) [17], which conceptualizes health care utilization as the result of interactions among predisposing characteristics, enabling resources, and need factors, situated within broader health system and contextual environments.

In the expanded model:

- Predisposing factors include demographic characteristics (e.g., age, education), social structure (e.g., socioeconomic position, cultural norms), and health-related beliefs that influence an individual’s propensity to use services.
- Enabling factors refer to individual and contextual resources that facilitate or constrain access, including income, health insurance, service availability, referral pathways, and organizational arrangements within the health care system.
- Need factors comprise both perceived need (self-identified symptoms or oral discomfort) and evaluated need (clinically assessed conditions), which represent the most immediate determinants of service use.

Unlike cognition-focused behavioral models, Andersen’s framework emphasizes structural, social, and health system determinants alongside individual characteristics in explaining patterns of health care utilization.

#### Application to oral healthcare utilization during pregnancy

In this mixed-methods review, Andersen’s model was used as an interpretive analytical framework following inductive thematic synthesis. Initial coding of qualitative findings and extraction of quantitative determinants were conducted without imposing an a priori theoretical structure. Emerging themes were subsequently deductively mapped onto Andersen’s domains to provide conceptual organization and explanatory coherence.

Within this framework:

- Misconceptions regarding the safety of dental treatment during pregnancy, educational level, and cultural norms surrounding pregnancy care were categorized as predisposing factors.
- Financial constraints, lack of dental insurance, absence of antenatal referral systems, provider hesitancy, and health system fragmentation were mapped as enabling factors.
- Pain, gingival bleeding, visible oral disease, and perceived oral health problems were classified as need factors, often triggering reactive rather than preventive care-seeking.

#### Operationalization in the synthesis

The model was operationalized at the interpretive stage of synthesis rather than as a statistical explanatory model. After inductive development of themes, findings were organized under Andersen’s domains to:

- Structure heterogeneous evidence across qualitative and quantitative study designs.
- Identify multilevel determinants operating at individual, interpersonal, and system levels.
- Examine whether oral health care utilization during pregnancy appeared primarily driven by enabling constraints, predisposing characteristics, or symptom-based need.

This theory-informed mapping enhanced conceptual clarity and strengthened policy-relevant interpretation while preserving the data-driven integrity of the synthesis.

## Results

### Search outcomes

A total of 1390 records were identified from database and 111 from citation search. After removing 390 duplicates, 920 records underwent title and abstract screening. Of these, 750 were excluded based on irrelevance to the review objectives. Full-text assessment was conducted on 170 articles, with 61 full texts unavailable. Among the 109 full-text articles reviewed, 47 satisfied the eligibility criteria. An additional 8 studies were identified through reference list screening, resulting in a final inclusion of 55 primary studies (Fig. 1).

### Study characteristics

The 55 included studies encompassed diverse methodological approaches: 37 quantitative, 17 qualitative, and 1 mixed-methods study published between 2005 and 2025 across 24 countries. The most represented countries were the USA (*n* = 10) [3, 15, 19, 30–36], Australia (*n* = 6) [20, 21, 37–40], India (*n* = 5) [41–45], and Canada (*n* = 4) [46–49].

Quantitative studies predominantly used cross-sectional (*n* = 35), prevalence (*n* = 6), and cohort (*n* = 1) designs (Table 1). Qualitative studies employed descriptive methodologies, primarily using semi-structured interviews (*n* = 14) and focus group discussions (*n* = 3) (Table 1). A mixed-methods study utilized cross-sectional survey and qualitative interviews [46] (Table 1). Sample sizes ranged from 10 participants [39] to over 145,000 [15].

**Table 1:** Characteristics of Included study

Author/year	Country	Study design	Age	Setting	Sample size	Trimester	Data collection	Outcome
**Quantitative study**								
Hullah et al./2007 [50]#	United -Kingdom	Prevalence study	NS	Hospital-based	206	Postnatal within 3 days of delivery	Questionnaire	Barriers-Unawareness of free oral care (26%)
Rafeek et al./2020 [51] #	Trinidad and Tobago	Prevalence study	15–46 and above	Maternity Hospital	161	All three-trimester	Questionnaire and Oral Health Examination	No routine oral care—148 (91.9%),Cost of dental treatment −88(54.7%),
Timothe et al./2005 [19] #	United States	Analytical cross-sectional study	18–44	Community-based, urban and rural level	4619	NS	Structured telephone interviews	Barriers-lower household income(29%)
Amin and ElSalhy/2014 [49] #	Canada	Prevalence study	NS	Hospital-based	423	NS	Structured questionnaire	Facilitators–Awareness of oral health–pregnancy link(p < 0.001)
Sekele et al./2025 [52] #	Democratic Republic of Congo	Prevalence study	18 years and above	Hospital-based	500	NS	Structured questionnaire	Barriers-Unawareness 423 (84.6%)
Hebbal et al./2025 [53] #	Saudi Arabia	Prevalence study	Less than 20-More than 35	Hospitals (Government and Private)	1120	All three-trimester	Structured questionnaire	Barriers-Lack of guidance from healthcare professionals (15.1%)
Dinas et al./2007 [54] #	Greece	Prevalence study	NS	Hospital-based	425	postpartum	Questionnaire	Barriers-Lack of education, Concern about negative effects (72.2)
Javali et al./2022 [41] #	India	Prevalence study	18–38	Hospital-based	445	All three-trimester	Questionnaire	Facilitator-Belief that routine dental check-ups are necessary during pregnancy(95.5)
Ali et al./2018 [55] #	Pakistan	Prevalence study	NS	Hospital -based, urban	570	NS	Questionnaire	Barriers-Ignored oral care (50.2), Fear of dental treatments (30.4%)
Russell et al./2021 [36] #	United states	Prevalence study	Above 18	Hospital-based	298	All three-trimester	Self-administered questionnaire	Facilitator-Referral by prenatal care provider (72.5)
Lafebre -Carrasco et al./2022 [56] #	Ecuador	Prevalence study	14–46	Hospital -based	1971	NS	Questionnaire	Barrier-Believed oral care is not safe (65.5%)
Bhagat et al./2022 [57] #	Nepal	Prevalence study	17–46	Hospital-based	600	All three-trimester	Structured questionnaire	Barriers-Time deficit (29.2), safety issues (14.8%). Facilitators-Positive outlook towards oral care (62.2)
Jain et al./2021 [42] #	India	Prevalence study	15–44	Hospital-based	380	NS	Questionnaire	Barriers-Family pressure (14.7%), Safety concern of local anaesthesia (14.8)
Gao et al./2021 [38] #	Australia	Prevalence study	NS	Hospital-based	427	NS	Questionnaire	Facilitators-Importance for oral health (88.3%)
Sun et al./2014 [58] #	China	Prevalence study	Under 20–36 and older	Hospital- based	2259	NS	Questionnaire	Barriers-Lower age (16.15%), Limited income (9.68%)
Saddki et al./2010 [59] #	Malaysia	Prevalence study	Less than 20–40 and above	Hospital-based	124	2nd trimester	Self-administered questionnaire	Barriers-Neglect of oral health(65.9%),Prolonged waiting at dental clinics (71.6)
Subedi et al./2024 [60] #	Nepal	Prevalence study	18–35	Hospital-based	139	All three-trimester	Semi-structured questionnaire	Barriers-Lacking awareness (48.9%), Deficient guidance (46.2%)
Onwuka et al./2021 [61] #	Nigeria	Prevalence study	20–40 and above	Hospital-based, urban	413	NS	Semi-structured questionnaire	Barriers-Place of residence (84.5), Occupation (*p* = 0.019), Oral health practices (< 0.01)
Rahebi et al./2021 [62] #	Iran	Prevalence study	15–35 and above	Community-based, rural and urban	4071	NS	Interview, researcher-made checklists, household health records	Barriers-Less care in rural areas was a barrier (13.3%,)
Habashneh et al./2005 [3] #	United states	Prevalence study	20 More than 36	Community -based	625	NS	Self-administered mailed questionnaire	Barriers-Regular dental check-up (*p* < 0.0001), Insurance coverage (*p* < 0.0001)
Sattar and Khan./2020 [63] #	Pakistan	Prevalence study	14–40	Hospital-based,Urban	183	All three-trimester	Structured questionnaire	Facilitators-Education is a facilitator (*p* < 0.05)
Riaz et al./2020 [64] #	Pakistan	Prevalence study	NS	Hospital-based,urban	260	All three-trimester	Questionnaire	Facilitators-Provision of Oral Health Information(42.8%)
Lee et al./2024 [32] #	United states	Prevalence study	20–35 and above	Community-based, rural and urban	62,189	NS	Questionnaire	Barriers- Fear of harm during treatment (*p* < 0.001) Facilitators-Dental Insurance improved dental attendance (*p* < 0.001)
Baskaradoss et al./2020 [43] #	India	Analytical cross sectional study	18–35	Hospital-based, urban	450	1 st trimester	Structured questionnaire	Barriers-Low level literacy (33.7%), Poor self-perception (40.5%)
Robison et al./2021 [15] #	United states	Prevalence study	Less than 19 to more than 35	Hospital-based, urban	145,051	All three-trimester	Structured questionnaire	Barriers-No dental insurance coverage (43.3%),low dental attendance (27.6%)
Naavaal et al./2019 [33] #	United states	Analytical cross-sectional study	Less than 19–35 and above	Community-based	1344	NS	Structured questionnaire	Facilitators-Dental insurance coverage (77%), Attention to oral care (57%)
Albasry et al./2019 [65] #	Saudi Arabia	Prevalence study	NS	Hospital- based, urban	270	All three -trimester	Self-administered questionnaire	Barriers-Safety concern (58.3%)
Barman et al./2019 [44] #	India	Prevalence study	18–37	Rural/urban	300	NS	interviews and pre tested questionnaire	Barriers-Impact of unemployment (*p* < 0.0001), Access to oral care (*p* < 0.0001)
Azab et al./2024 [66] #	Saudi Arabia	Analytical cross- sectional study	18–48	Hospital-based, urban	361	All three-trimester	Self-administered questionnaire	Barriers-Lack of knowledge on safety (31.57%), Financial barriers (30.2%)
Bhaskar et al./2020 [45]#	India	Prevalence study	Less than 24- more than 31	Hospital-based	400	All three-trimester	Interview-based structured questionnaire	Barriers-Low concern for oral health was a barrier (59.4%)
Kaba et al./2022 [67] #	Kenya	Prevalence study	NS	Hospital based	309	All three-trimester	Interviewer-administered structured questionnaires	Barriers-Lack of information (83%),Faulty belief (94%)
Honkala et al./2005 [68] #	Kuwait	Prevalence study	18–56 years	Hospital based, urban	603	NS	Structured questionnaire	Facilitators-Parity (57%)
Al -Swuailem et al./2013 [69] #	Saudi Arabia	Analytical cross-sectional study	18–48 years	Hospital based, urban	959	NS	Self-administered questionnaire	Facilitators-Benefits of previous dental visits (*p* < 0.001)
Boggess et al./2010 [34]#	United states	Prevalence study	18–36 and above	Hospital based, urban	599	All three-trimester	Self-administered questionnaire	Barriers-Lack of receiving routine dental (35%)
Gupta and Chhetry/2019 [70] #	Nepal	Prevalence study	20–30 and above	Hospital based, urban	50	3rd trimester	Interview using a pre-designed proforma	Barriers-Low priority (48%)
George et al./2013 [37] #	Australia	Prevalence study	NS	Hospital based, urban	241	All three-trimester	Structured questionnaire	Barriers-Less priority for oral health (32%); Facilitators-high household income (69.4%.)
Al Jallad et al./2022 [35] #	United States	Cohort study	18–30 and more	Hospital-based	186	NS	Questionnaire, Record, oral examination	Facilitators-Previous dental visits increased prenatal visits (64.3)
**Qualitative study**								
Kong et al./2021 [20]	Australia	Descriptive qualitative study	18–36	Community-based	12	All three trimester	Telephone or face-to-face interviews	Barriers-policy barriers; Facilitator-Reduction in fees
Porras et al./2024 [71]	Columbia	Phenomenological Study	18–45	Community-based	24	2nd and 3rd trimester	Semi-structured interviews	Barriers Safety and quality of oral care
Bahramian et al./2018 [11]	Iran	Descriptive qualitative study	NS	Community based, urban	22	NS	Semi -structured interviews and FGD	Barriers-Lack of awareness, misconception
Adeniyi et al./2021 [47]	Canada	Descriptive qualitative study	25–40	Community-based, Urban	14	All three trimester	Semi-structured interviews	Barriers-Negative attitude to oral care
Wilson et al./2023 [21]	Australia	Descriptive qualitative study	NS	Community-based	15	All three trimester	Semi-structured interviews	Barriers-Perceived importance of oral healthcare, Policy Barriers
Le et al./2009 [31]	United states	Descriptive qualitative study	NS	Community-based	51	NS	Semi-structured telephone interviews	Barriers-safety concern;Facilitators-Good communication of dentist
Al Khamis et al./2016 [72]	Kuwait	Descriptive qualitative study	19–42	Community-based, Urban	19	NS	In-depth interview	Barriers- Cultural beliefs
Adeniyi et al./2020 [47]	Canada	Descriptive qualitative study	21–41	Hospital-based	17	NS	Focus Group Discussions	Barriers-lack of interprofessional antenatal collaboration
Wang et al./2020 [73]	China	Phenomenological Qualitative Study	27–32	Hospital-based, urban	317	All three trimester	Focus Group Discussions	Barriers-Competing Priorities, Lack of awareness
Buerlein et al./2011 [74]	China	Descriptive qualitative study	NS	Community-based, rural and urban	34	NS	Focus Group Discussions	Barriers-Fear/anxiety, Lack of insurance coverage
Winckler et al./2024 [75]	Denmark	Descriptive qualitative study	29 (20–42)	Hospital based, urban	23	1 st trimester	Structured paper-based questionnaire	Barriers-Cost of dental treatment
Phoosuwan et al./2024 [76]	Thailand	Descriptive qualitative study	18–43	Community -based	20	1 st and 2nd trimester	Semi-structured interviews	Barriers-Wrong information, Fear/anxiety
Vamos et al./2019 [30]	United states	Descriptive qualitative Study	19–43	Hospital-based, urban	17	NS	Focus Group Discussions	Barriers- Fear/anxiety;Facilitator-Child’s wellbeing
Fakheran et al./2020 [77]	Iran	Descriptive qualitative study	18–40	Hospital-based, urban	27	All three trimester	Semi-structured face-to-face interviews	Barriers- Safety concern, Cost of dental treatment
George et al./2012 [39]	Australia	Descriptive qualitative study	18–30	Hospital-based, urban	10	All three trimester	Semi-structured telephone interviews	Barriers-Wrong information, Fear/anxiety
Riggs et al./2016 [40]	Australia	Descriptive qualitative study	NS	Hospital, community-based, urban	24	NS	Focus Group Discussions	Barriers- Cultural beliefs, Lack of support from Healthcare providers
Liu et al./2019 [78]	Hong Kong	Descriptive qualitative study	NS	Hospital-based	30	3rd trimester	Semi-structured interviews	Barriers-Misconception; Facilitators-Education by healthcare provider
**Mixed-Method Study**								
Kamalabadi et al./2024[46]	Canada	Mixed-Method study Quantitative-Analytical crosssectional Qualitative descriptive	18–43	Hospital-based	130	All three trimester	Self-administered questionnaire	Barriers-Misconception, Foetal safety (48.2%)

Quantitative findings from all included quantitative studies were transformed into qualitative themes using a convergent integrated approach

*NS* not specified

### Methodological quality

The included studies demonstrated moderate to high methodological quality based on the JBI critical appraisal tools. This was interpreted descriptively without applying rigid numerical cut-offs. Prevalence studies (Table 2) averaged a score of 16.9/18, while analytical cross-sectional studies (Table 3) scored 14.8/16. The cohort study (Table 4) achieved a perfect score of 22/22. Qualitative studies (Table 5) scored an average of 17.1/20, though many lacked details regarding researcher reflexivity, positionality, and philosophical underpinning. The mixed-methods study [46] (Table 6) scored 14/18 (quantitative) and 18/20 (qualitative). Common limitations included use of non-validated tools in quantitative studies and inadequate reporting of epistemological frameworks in qualitative studies. The results of the critical appraisal informed the interpretation of the synthesis, with findings from studies with methodological limitations interpreted cautiously.

**Table 2:** Critical appraisal of prevalence study

Study	1	2	3	4	5	6	7	8	9	Score
Hullah et al./2007 [50]	Y	U	Y	Y	Y	Y	Y	N	U	14
Rafeek et al./2020 [51]	Y	N	N	Y	Y	Y	Y	Y	Y	14
LETimothe et al./2005 [19]	Y	Y	Y	Y	Y	Y	Y	Y	Y	18
Amin and ElSalhy/2014 [49]	Y	Y	Y	Y	Y	Y	Y	Y	Y	18
Sekele et al./2025 [52]	Y	Y	Y	Y	Y	Y	Y	Y	Y	18
Hebbal et al./2025 [53]	Y	Y	Y	Y	Y	Y	Y	Y	Y	18
Dinas et al./2007 [54]	Y	Y	Y	Y	Y	Y	Y	Y	Y	18
Javali et al./2022 [41]	Y	N	Y	Y	Y	N	N	Y	Y	12
Ali et al./2018 [55]	Y	Y	Y	Y	Y	Y	Y	Y	Y	18
Russell et al./2021 [36]	Y	Y	Y	Y	Y	Y	Y	Y	Y	18
Lafebre-Carrasco et al./202 [56]	Y	Y	Y	Y	Y	Y	Y	Y	Y	18
Bhagat et al./2022 [57]	Y	U	Y	Y	Y	Y	Y	Y	U	16
Jain et al./2021 [42]	Y	Y	Y	Y	Y	Y	Y	Y	Y	18
Gao et al./2021 [38]	Y	Y	Y	Y	Y	Y	Y	Y	Y	18
Sun et al./2014 [58]	Y	Y	Y	Y	Y	Y	Y	Y	Y	18
Saddki et al./2010 [59]	Y	N	N	Y	Y	Y	Y	Y	Y	14
Subedi et al./2024 [60]	Y	Y	Y	Y	Y	Y	Y	Y	Y	18
Onwuka et al./2021 [61]	Y	Y	Y	Y	Y	Y	Y	Y	U	17
Rahebi et al./2021 [62]	Y	Y	Y	Y	Y	Y	Y	Y	Y	18
Habashneh et al./2005 [3]	Y	Y	Y	Y	Y	Y	Y	N	Y	16
Sattar and Khan/2020 [63]	Y	Y	Y	Y	Y	Y	Y	Y	Y	18
Riaz et al./2020 [64]	Y	Y	Y	Y	Y	Y	Y	Y	Y	18
Lee et al./2024 [32]	Y	Y	Y	Y	Y	Y	Y	Y	Y	18
Robison et al./2021 [15]	Y	Y	Y	Y	Y	Y	Y	Y	Y	18
Albasry et al./2019 [65]	Y	Y	Y	Y	Y	Y	Y	Y	Y	18
Barman et al./2019 [44]	Y	Y	Y	Y	Y	Y	Y	Y	N	16
Bhaskar et al./2020 [45]	Y	Y	Y	Y	Y	Y	Y	Y	Y	18
Kaba et al./2022 [67]	Y	Y	Y	Y	Y	Y	Y	Y	Y	18
Honkala et al./2005 [68]	Y	Y	Y	Y	Y	U	Y	Y	Y	16
Boggess et al./2010 [34]	Y	Y	Y	Y	Y	Y	Y	Y	Y	18
Gupta and Chhetry/2019 [70]	N	U	U	Y	N	Y	Y	Y	Y	18
George et al./2013 [37]	Y	Y	Y	Y	Y	Y	Y	Y	Y	12

*Y* Yes, *N* No, *U* unclear, *N*/*a* not applicable

(1) Was the sample frame appropriate to address the target population? (2) Were study participants sampled in an appropriate way? (3) Was the sample size adequate? (4) Were the study subjects and the setting described in detail? (5) Was the data analysis conducted with sufficient coverage of the identified sample? (6) Were valid methods used for the identification of the condition? (7) Was the condition measured in a standard, reliable way for all participants? (8) Was there appropriate statistical analysis? (9) Was the response rate adequate, and if not, was the low response rate managed appropriately?

**Table 3:** Critical appraisal of analytical study

Study	1	2	3	4	5	6	7	8	Score
Timothe et al./2005 [19]	Y	Y	U	Y	U	Y	Y	Y	14
Baskaradoss et al./2020 [43]	Y	Y	Y	Y	Y	Y	Y	Y	16
Naavaal et al./2019 [33]	Y	Y	Y	Y	Y	Y	Y	Y	16
Azab et al./2024 [66]	Y	Y	U	U	Y	Y	Y	Y	14
Al-Swuailem et al./2013 [69]	Y	Y	Y	Y	Y	Y	Y	Y	14
%	100	100	60	80	80	100	100	100	

*Y* Yes, *N* No, *U* unclear, *N*/*a* not applicable

(1) Were the criteria for inclusion in the sample clearly defined? (2) Were the study subjects and the setting described in detail? (3) Was the exposure measured in a valid and reliable way? (4) Were objective, standard criteria used for measurement of the condition? (5) Were confounding factors identified? (6) Were strategies to deal with confounding factors stated? (7) Were the outcomes measured in a valid and reliable way? (8) Was appropriate statistical analysis used?

**Table 4:** Critical appraisal of cohort study

Study	1	2	3	4	5	6	7	8	9	10	11	Score
Al Jallad et al./2022 [35]	Y	Y	Y	Y	Y	Y	Y	Y	Y	Y	Y	22
%	100	100	100	100	100	100	100	100	100	100	100	

*Y* Yes, *N* No, *U* unclear, *N*/*a* not applicable

(1) Were the two groups similar and recruited from the same population? (2) Were the exposures measured similarly to assign people to both exposed and unexposed groups? (3) Was the exposure measured in a valid and reliable way? (4) Were confounding factors identified? (5) Were strategies to deal with confounding factors stated? (6) Were the groups/participants free of the outcome at the start of the study (or at the moment of exposure)? (7) Were the outcomes measured in a valid and reliable way? (8) Was the follow up time reported and sufficient to be long enough for outcomes to occur? (9) Was follow up complete, and if not, were the reasons to loss to follow up described and explored? (10) Were strategies to address incomplete follow up utilized? (11) Was appropriate statistical analysis used?

**Table 5:** Critical appraisal of qualitative study

Study	1	2	3	4	5	6	7	8	9	10	Score
Kong et al./2021 [20]	Y	Y	Y	Y	Y	Y	Y	Y	Y	Y	20
Porras et al./2024 [71]	Y	Y	Y	Y	Y	N	N	Y	Y	Y	16
Bahramian et al./2018 [11]	Y	Y	Y	Y	Y	N	N	Y	Y	Y	16
Adeniyi et al./2021 [47]	Y	Y	Y	Y	Y	N	N	Y	Y	Y	16
Wilson et al./2023 [21]	Y	Y	Y	Y	Y	Y	Y	Y	Y	Y	20
Le et al./2009 [31]	Y	Y	Y	Y	Y	N	Y	Y	Y	Y	18
Al Khamis et al./2016 [72]	Y	Y	Y	Y	Y	N	N	Y	Y	Y	16
Adeniyi et al./2020 [47]	Y	Y	Y	Y	Y	N	Y	Y	Y	Y	18
Wang et al./2020 [73]	Y	Y	Y	Y	Y	N	N	Y	Y	Y	16
Buerlein et al./2011 [74]	Y	Y	Y	Y	Y	N	N	Y	N	Y	14
Winckler et al./2024 [75]	Y	Y	Y	Y	Y	N/A	N/A	Y	Y	Y	16
Phoosuwan et al./2024 [76]	Y	Y	Y	Y	Y	N	N	Y	Y	Y	16
Vamos et al./2019 [30]	Y	Y	Y	Y	Y	N	N	Y	Y	Y	16
Fakheran et al./2020 [77]	Y	Y	Y	Y	Y	Y	Y	Y	Y	Y	20
George et al./2012 [39]	Y	Y	Y	Y	Y	U	U	Y	Y	Y	18
Riggs et al./2016 [40]	Y	Y	Y	Y	Y	N	Y	Y	Y	Y	18
Liu et al./2019 [78]	Y	Y	Y	Y	Y	N	N	Y	Y	Y	16
%	100	100	100	100	100	21.05	31.57	100	94.73	100	

*Y* Yes, *N* No, *U* unclear, *N*/*a* not applicable

(1) Is there congruity between the stated philosophical perspective and the research methodology? (2) Is there congruity between the research methodology and the research question or objectives? (3) Is there congruity between the research methodology and the methods used to collect data? (4) Is there congruity between the research methodology and the representation and analysis of data? (5) Is there congruity between the research methodology and the interpretation of results? (6) Is there a statement locating the researcher culturally or theoretically? (7) Is the influence of the researcher on the research, and vice-versa, addressed? (8) Are participants, and their voices, adequately represented? (9) Is the research ethical according to current criteria or, for recent studies, and is there evidence of ethical approval by an appropriate body? (10) Do the conclusions drawn in the research report flow from the analysis, or interpretation, of the data?

**Table 6:** Critical appraisal of Mixed-Method study

**Study**	**1**	**2**	**3**	**4**	**5**	**6**	**7**	**8**	**9**		**Score**
Amalabadi et al./2024 [46]	Y	U	Y	Y	Y	U	Y	Y	Y		14
	1	2	3	4	5	6	7	8	9	10	Score
	Y	Y	Y	Y	Y	U	U	Y	Y	Y	18

*Y* Yes, *N* No, *U* unclear, *N*/*a* not applicable

(1) Were the criteria for inclusion in the sample clearly defined? (2) Were the study subjects and the setting described in detail? (3) Was the exposure measured in a valid and reliable way? (4) Were objective, standard criteria used for measurement of the condition? (5) Were confounding factors identified? (6) Were strategies to deal with confounding factors stated? (7) Were the outcomes measured in a valid and reliable way? (8) Was appropriate statistical analysis used?

(1) Is there congruity between the stated philosophical perspective and the research methodology? (2) Is there congruity between the research methodology and the research question or objectives? (3) Is there congruity between the research methodology and the methods used to collect data? (4) Is there congruity between the research methodology and the representation and analysis of data? (5) Is there congruity between the research methodology and the interpretation of results? (6) Is there a statement locating the researcher culturally or theoretically? (7) Is the influence of the researcher on the research, and vice-versa, addressed? (8) Are participants, and their voices, adequately represented? (9) Is the research ethical according to current criteria or, for recent studies, and is there evidence of ethical approval by an appropriate body? (10) Do the conclusions drawn in the research report flow from the analysis, or interpretation, of the data?

### Synthesized findings

The synthesized findings are presented in Fig. 3, which illustrates the integration of qualitative themes and qualitized quantitative findings within the domains of Andersen’s Behavioral Model. In this review, “qualitized” findings refer to quantitative results (e.g., statistically significant associations and prevalence patterns) that were transformed into narrative statements to enable integration with qualitative data, following JBI’s convergent integrated approach [22]. Quantitative findings were extracted from cross-sectional, prevalence, and cohort studies included in this review. The figure represents a conceptual framework illustrating how individual, contextual, and need-related factors interact to influence oral health care utilization during pregnancy.

Based on the Andersen’s Behavioral Model of Health Services utilization, this mixed-method systematic review identified the following barriers and facilitators of oral care utilization among pregnant women (Fig. 3):

1. Barriers to use of oral health services in pregnancy (Individual Predisposing Factors)
  This synthesis included 33 primary studies contributing 27 qualitative and 13 qualitized quantitative findings. Quantitative evidence was mainly from cross-sectional and prevalence surveys, while qualitative data were derived from interviews and focus groups across diverse settings. Overall, methodological quality was moderate to high; however, some quantitative studies relied on self-reported, non-validated measures, and several qualitative studies did not clearly report reflexivity.
  Many pregnant women perceived oral health care as unnecessary during pregnancy or feared that dental procedures (e.g., X-rays, anesthesia, scaling) might harm the fetus. These beliefs were often fueled by misinformation, cultural taboos, or lack of prior dental education. This fear-based avoidance was reinforced by negative past experiences and low oral health literacy (Fig. 3).
2. Barriers from health care system and social security (Contextual Enabling Factors)
  This synthesis included 29 primary studies contributing 25 qualitative and 10 qualitized quantitative findings. Evidence was mainly derived from cross-sectional surveys and interview-based studies across diverse health care systems. Methodological quality was generally moderate to high, although several studies relied on self-reported financial and insurance-related barriers.
  Limited dental insurance coverage, high out-of-pocket costs, and the absence of subsidized oral health services for pregnant women were major deterrents. A subset of included studies reported lower utilization among minority, migrant, and rural populations; however, detailed sociodemographic disaggregation was inconsistently reported across studies. Lack of referral from antenatal care providers further constrained access (Fig. 3a).
3. Barriers due to health care behavior (Health Behavior Factors)
  This synthesis included 35 primary studies contributing 41 qualitative and 24 qualitized quantitative findings. Evidence was largely derived from cross-sectional surveys and interview-based studies. Methodological quality was generally moderate to high, although several studies relied on self-reported behavioral measures.
  Oral health was deprioritized in the context of pregnancy-related fatigue, work obligations, caregiving duties, and logistical challenges. Many women reported not seeking oral care unless symptoms became severe, reflecting a reactive rather than preventive approach to oral health (Fig. 3b).
4. Barriers to oral health care availability in pregnancy (Contextual Enabling Factors)
  This synthesis included 12 primary studies contributing 11 qualitative and 1 qualitized quantitative finding. Evidence was largely derived from interview-based studies across varied health care settings. Overall methodological quality was moderate to high, although some qualitative studies did not fully report reflexivity.
  Reluctance among dentists to treat pregnant women due to perceived medicolegal risk or lack of training in gestational care was noted. Similarly, antenatal care (ANC) providers often lacked the knowledge or protocols to advise patients on oral health, reflecting systemic under integration of dental services in maternal care frameworks (Fig. 3c).
5. Barriers due to cultural factors (Individual Predisposing Factors)
  This synthesis included 15 primary studies contributing 11 qualitative and 7 qualitized quantitative findings. Evidence was derived from cross-sectional surveys and interview-based studies across diverse cultural contexts. Methodological quality was generally moderate to high, though several studies relied on self-reported belief measures.
  In several cultural contexts, pregnancy was seen as a vulnerable period during which medical interventions including oral care should be avoided. These norms were often reinforced by family members, elders, or community influencers, limiting women’s autonomy in health-seeking (Fig. 3d).
6. Facilitators for seeking oral care in pregnancy (Individual Enabling Factors)
  This synthesis included 26 primary studies, contributing 9 qualitative and 24 qualitized quantitative findings. Evidence was predominantly derived from cross-sectional surveys assessing socioeconomic and educational determinants. Overall methodological quality was moderate to high, although most quantitative studies relied on self-reported utilization measures.
  Higher levels of education, employment status, urban residence, and prior positive dental experiences were consistently associated with greater oral health care utilization. Women with better oral health literacy were more likely to seek preventive care during pregnancy (Fig. 3e).
7. Facilitators related to perceived health status (Individual Need Factors)
  This synthesis included 8 primary studies contributing 2 qualitative and 4 qualitized quantitative findings. Evidence was primarily derived from cross-sectional surveys assessing symptom-driven care-seeking behavior. Methodological quality was generally moderate to high, though reliance on self-reported symptom perception may introduce reporting bias.
  Recognition of visible or painful symptoms (e.g., gingival bleeding, toothache, swelling) prompted care-seeking. However, reliance on symptom perception meant many women delayed treatment until conditions worsened, missing preventive opportunities (Fig. 2f).

**Fig. 2:**
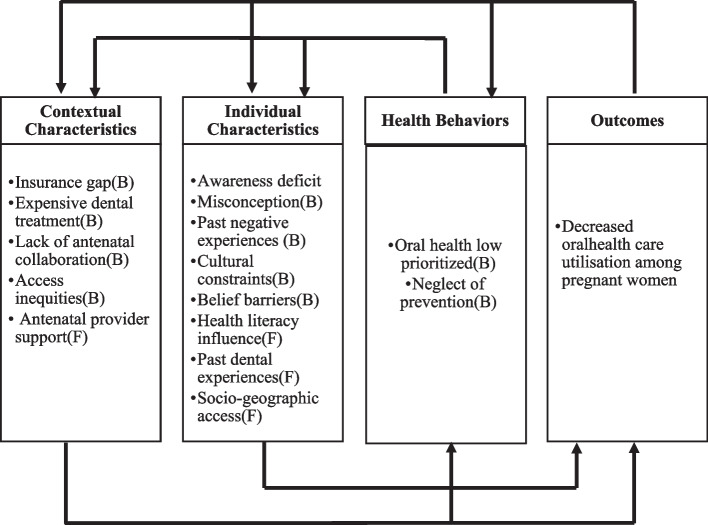
Summary of findings—barierrs and facilitators for the utilization of dental care service among pregnant women according to the Anderson behavioral model. *B* barrier, *F* facilitators
8. Facilitators from health care system for seeking oral health care in pregnancy (Contextual Enabling Factors)
  This synthesis included 17 primary studies contributing 11 qualitative and 3 qualitized quantitative findings. Evidence was derived from interview-based studies and cross-sectional surveys examining provider guidance and referral practices. Overall methodological quality was moderate to high, although most quantitative studies relied on self-reported utilization measures.
  ANC provider support emerged as a strong enabler. Women were significantly more likely to seek oral care when they received clear guidance, reassurance, and referrals from trusted obstetric or primary care providers (Fig. 3g).

**Fig. 3:**
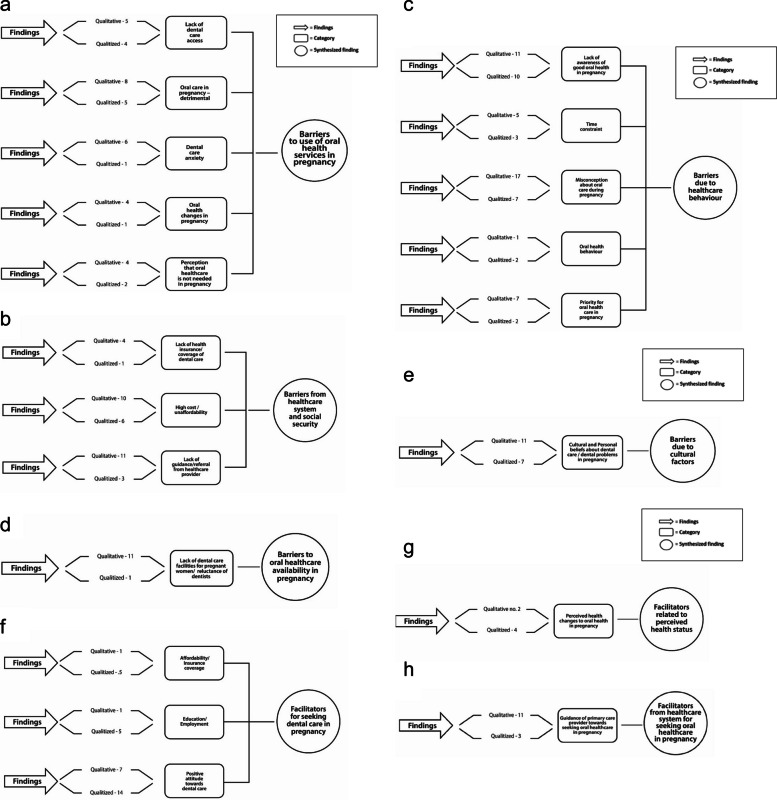
**a** Barriers to the use of oral health services during pregnancy. **b** Barriers from healthcare system and social security. **c** Barriers due to healthcare behavior. **d** Barriers to oral healthcare availability in pregnancy. **e** Barriers due to cultural factors. **f** Facilitators for seeking dental care in pregnancy. **g** Facilitators related to perceived health status. **h** Facilitators from healthcare system for seeking oral healthcare in pregnancy

## Discussion

This mixed-methods systematic review was undertaken to identify the barriers and facilitators of oral health care utilization among pregnant women. Quantitative, qualitative, and mixed-method studies were critically appraised and synthesized, with findings organized into eight categories using Andersen’s Behavioral Model of Health Services Utilization [17].

While previous studies have examined either qualitative or quantitative aspects in isolation, no review had comprehensively integrated both forms of evidence. Our review addressed this gap by combining global prevalence data with rich qualitative narratives, thereby offering a more complete understanding of maternal oral health behaviors. These holistic findings strengthen the case for evidence-based, culturally sensitive, and system-level strategies to improve access to oral health care during pregnancy.

Guided by the JBI methodology for mixed methods systematic reviews [22], we were able to merge measurable patterns with contextual determinants such as fear, stigma, misinformation, cultural taboos, and perceived vulnerabilities. This integrative approach provided insights that neither quantitative nor thematic synthesis of qualitative evidence alone could achieve. Building on earlier qualitative syntheses [10, 11], this review adds value by associating global prevalence patterns with individual-level experiences.

An insight into the review findings based on Andersen’s Behavioral Model is as follows:

1. Perceptions, misinformation, and fear: A persistent theme across included studies was the misconception that dental procedures such as X-rays, anaesthesia, or extractions pose risks to fetal health. These beliefs are widespread and deeply rooted in sociocultural narratives, as confirmed by studies from different countries like Iran [11], Denmark [75], Nepal [60], India [42], and Australia [40]. In many cultures, pregnancy is seen as a sacred state when treatments should be avoided. Similar findings were reported by Kamalabadi et al. [12] who identified unfavorable beliefs and oral health misinformation as leading barriers to seeking oral care in pregnancy. This review reaffirms that misinformation is not only prevalent but persistent, transcending geographic and economic boundaries.
2. Economic and systemic constraints: Other major barriers to dental services utilization in pregnancy were lack of dental insurance, high cost of services, and insufficient availability of pregnancy-safe oral care settings. Severity of these financial constraints worsens in low- and middle-income countries. In these settings, oral health care is often not included in routine antenatal services and is delivered predominantly through the private sector [16, 61]. Interestingly, even in high-income settings such as the USA and Canada, minority and immigrant women frequently face cost-related barriers, indicating that universal health care access does not automatically translate into equitable utilization [15, 19, 31, 46, 49]. Affordability and insurance coverage for dental treatments favored health seeking behaviors as demonstrated by Robinson et al. [15] and Naavaal et al. [33].
  Additionally, reluctance among dentists to treat pregnant women due to concerns over legal liability or lack of training, as reported worsens these access issues [16, 31]. Similarly, lack of interprofessional collaboration and negative attitude of other health care personnel like gynecologists, midwives, and physicians toward oral care in pregnancy was a barrier [11, 16, 31]. Our review findings strongly advocate for integrated antenatal-dental service models supported by provider training and regulatory guidance.
3. Time constraints, competing responsibilities, and behavior: Several studies included in the review highlighted those pregnant women, especially those with multiple caregiving or work responsibilities, deprioritized oral care due to time constraints or perceived lack of urgency. This finding aligns with multiple studies reporting that fatigue, childcare, and work-related duties often take precedence during pregnancy [11, 20, 75]. These suggest a low priority for dental well-being, thereby resorting to dental treatment only in pain or similar emergencies. It shows that oral health services is often framed as curative rather than preventive. Behavioral change strategies, especially those employing motivational interviewing and personalized risk communication, may help shift this perception.
4. Cultural norms and social determinants: Cultural taboos and social norms play a profound role in shaping oral health care behavior. In several settings, women reported that family members discouraged oral care, viewing it as unnecessary or dangerous during pregnancy [11, 38, 69, 75]. This aligns with the concept of “social permission” in health-seeking, where community beliefs mediate individual agency [79]. The present review further confirms that interventions must engage not only individual women but also their families and communities to be effective. Cultural adaptation of educational content and involvement of community influencers, such as midwives or religious leaders, can enhance acceptability and uptake.
  Higher education levels, employment, and urban residence consistently emerged as facilitators. These findings mirror studies by Robison et al. [15], George et al. [37], and Lee et al. [80], who found that oral health literacy, empowerment, and prior positive dental experiences significantly enhance care-seeking behavior.
5. Facilitators from providers and perceived health need: When women perceived changes in oral health such as gum bleeding or pain, they were more likely to seek care. This finding is consistent with Andersen’s Behavioral Model of Health Services Use [17, 18] and with previous studies showing that symptom perception is a critical motivator for care [10, 80]. Antenatal care providers’ encouragement emerged as one of the strongest facilitators in our review. Similar findings were reported by Wilson et al. [21] and Naavaal et al. [33], who found that verbal referrals and structured dental education from these health care personnel significantly increased dental visits [15, 33]. This highlights potential of ANC providers as catalysts for behavior change and consequently lead to inclusion of oral health modules in medical and nursing education.

## Strengths and limitations

This review possesses several strengths that enhance its rigor, relevance, and potential impact. To our knowledge, this is the first mixed-methods systematic review to synthesize both quantitative and qualitative evidence on barriers and facilitators of oral health care utilization among pregnant women. The use of a convergent integrated approach enabled transformation and synthesis of diverse forms of evidence, yielding richer and more comprehensive insights than single-method reviews.

The review adhered to PRISMA guidelines for transparent reporting and incorporated extensive searches across multiple databases and grey literature sources. Inclusion of studies from diverse geographic, cultural, and economic contexts provided a broad global perspective. Additionally, the application of Andersen’s Behavioral Model as a theoretical framework offered a structured and conceptually grounded lens to interpret cross-level determinants of care-seeking behavior. The focus on pregnant women lived experiences ensured a patient-centered perspective, strengthening the relevance of findings for equitable and culturally appropriate policy and practice.

However, several limitations should be acknowledged. Restriction to English-language publications introduces a potential risk of language bias and may have contributed to geographic skew, particularly given that oral health research from low- and middle-income countries is often published in local languages. Consequently, certain context-specific insights may be underrepresented.

Additionally, the exclusion of high-risk pregnancies (e.g., gestational diabetes, preeclampsia, and other comorbid conditions) may have enhanced internal consistency by reducing clinical heterogeneity. However, this decision may limit the external validity of the findings, as such conditions are relatively common and often coexist with poor oral health. Future research should examine utilization patterns among high-risk groups to ensure more inclusive and generalizable insights.

Although inclusion of both qualitative and quantitative studies enriched the synthesis, heterogeneity in study designs, outcome measures, and populations precluded meta-analysis and limited direct comparison of effect sizes across settings. Furthermore, many quantitative studies relied on self-reported or non-validated measures, raising concerns regarding recall bias and measurement validity. Some qualitative studies provided limited detail on reflexivity or researcher positionality, which may influence interpretive credibility. In line with methodological guidance for mixed-methods synthesis, qualitative findings were synthesized interpretatively rather than hierarchically weighted by checklist scores, to avoid reducing methodological diversity to a single numeric metric. Incorporating risk-of-bias assessments into interpretative weighting in mixed-methods reviews presents methodological challenges, as appraisal tools for qualitative and quantitative research assess different constructs of rigor and validity. As such, ROB findings were not used to differentially weight themes or quantitative associations. While this approach preserves methodological pluralism, it may limit the extent to which study quality influenced overall conclusions.

Finally, insufficient disaggregated reporting by sociodemographic variables such as race, caste, or migration status restricts deeper understanding of intersectional inequities in oral health care utilization.

### Implications for further research

This review shows the barriers for seeking oral health services in pregnancy at the individual, community, and system levels. These are the areas that require further research. Future studies should explore longitudinal patterns of oral health behavior across pregnancy and postpartum to identify critical windows for intervention. More research is needed on intersecting factors such as caste, race, disability, and rural residence compound barriers. Also, interventions on integrated care models involving dental-medical personnels should be evaluated for feasibility and cost-effectiveness.

Development of validated instruments to assess oral health literacy, beliefs, and behavior during pregnancy is an overlooked area that needs further attention. Such tools, particularly those grounded in behavioral theory, are critical for designing effective interventions.

### Implications for policy

Based on the synthesized findings of this review, several potential policy approaches may be considered to improve oral health care utilization during pregnancy. Integration of oral health into antenatal care should be prioritized within national maternal health policies. For example, Australia has incorporated oral health recommendations within national antenatal guidelines [81, 82] demonstrating policy-level commitment to integrating dental care within routine pregnancy care pathways. Similarly, in the USA, pilot interprofessional models integrating oral health education into prenatal care settings have demonstrated feasibility and improved patient engagement [83].

Insurance and Financial Coverage must prioritize oral care subsidies for pregnant women. Inclusion of oral health in national maternal health packages can alleviate financial barriers and promote equity. Interprofessional Training and Referral Systems can reduce provider hesitancy and promote interprofessional collaboration.

To enhance feasibility, specific policy instruments may include incorporating oral health screening into antenatal care quality indicators, introducing reimbursement codes for prenatal dental consultations within public and private insurance schemes, and mandating structured oral health training modules within obstetric and midwifery curricula. Establishing formal referral pathways such as integrated electronic referral systems between antenatal clinics and dental services may further strengthen continuity of care and reduce provider-level barriers.

## Conclusion

This mixed-methods systematic review, grounded in Andersen’s Behavioral Model of Health Services utilization, gives comprehensive and integrated insights into the multilevel barriers and facilitators influencing oral health care utilization among pregnant women. This review reveals a complex interplay of misinformation, systemic inaccessibility, cultural norms, and provider behaviors that collectively shape oral health decisions during pregnancy.

The review strengthens the existing evidence base by mapping these determinants within a well-established theoretical framework and identifying gaps existing in service integration and access. The findings are consistent with existing literature, highlighting the urgent need for integrated antenatal-dentalcare pathways, insurance reform that improve affordability and accessibility, interprofessional training, and culturally sensitive health education. Coordinated efforts across policy, clinical practice, research, and community engagement are essential to ensure equitable oral health care for pregnant women.

## Supplementary Information

Additional file 1.

## Data Availability

All data generated or analyzed during this systematic review are included in this published article and its additional files. The complete search strategies for all databases, data extraction forms, and quality appraisal results are provided as additional file. This review was prospectively registered in the PROSPERO database (ID: CRD42025642722; Date of registration: 05 March 2025). Additional datasets or materials related to this review are available from the corresponding author upon reasonable request.
