# Supplementary material for: Barriers and facilitators of oral health care utilization among pregnant women: a mixed-methods systematic review
Source: Syst Rev. 2026 Apr 20;15:182. doi: 10.1186/s13643-026-03151-8 (PMC13227648; doi:10.1186/s13643-026-03151-8)
Supplement: Supplementary file 1 — Additional file 1. [file 13643_2026_3151_MOESM1_ESM.docx]

**Additional file**

**Table 3 Search results in databases**

**3a. PubMed**

Date of search: 17/04/2025

| "Pregnant People"[Mesh] | 16,562 results |
| --- | --- |
| "Pregnant women" | 129,983 results |
| Pregnancy[Mesh] | 1,055,762 results |
| “Pregnant females” | 3,535 results |
| “Expecting mothers” | 167 results |
| “Expectant mothers” | 1,509 results |
| ((((("Pregnant People"[Mesh]) OR ("Pregnant women")) OR ("Pregnancy"[Mesh])) OR ("Pregnant females")) OR ("Expecting mothers")) OR ("Expectant mothers") | 1,082,963 results |
| Barriers | 493,869 results |
| Challenges | 1,455,847 results |
| Obstacles | 75,708 results |
| Hindrances | 15,739 results |
| Limitations | 2,503,473 results |
| Constraints | 138,462 results |
| Difficulties | 357,551 results |
| Facilitators | 827,786 results |
| Enablers | 732,609 results |
| Support | 12,168,228 results |
| (((((((((Barriers) OR (Challenges)) OR (Obstacles)) OR (Hindrances)) OR (Limitations)) OR (Constraints)) OR (Difficulties)) OR (Facilitators)) OR (Enablers)) OR (Support) | 15,356,153 results |
| "Oral healthcare utilization" | 24 results |
| "Oral healthcare utilisation" | 6 results |
| "Oral care utilization" | 9 results |
| "Oral care utilisation" | 1 result |
| "Dental care utilization" | 362 results |
| "Dental care utilisation" | 44 results |
| "Dental service utilization" | 216 results |
| "Dental service utilisation" | 49 results |
| "Dental Care"[Mesh] | 35,943 results |
| "Use of dental services" | 586 results |
| “Access to dental care" | 1,026 results |
| " Dental care accessibility" | 13 results |
| "Dental visits" | 2,244 results |
| "Dental attendance" | 692 results |
| "Dental Health Services"[Mesh] | 41,118 results |
| (((((((((((((("Oral healthcare utilization") OR ("Oral healthcare utilisation")) OR ("Oral care utilization")) OR ("Oral care utilisation")) OR ("Dental care utilization")) OR ("Dental care utilisation")) OR ("Dental service utilization")) OR ("Dental service utilisation")) OR ("Dental Care"[Mesh])) OR ("Use of dental services")) OR (" Dental care accessibility")) OR ("Access to dental care")) OR ("Dental visits")) OR ("Dental attendance")) OR ("Dental Health Services"[Mesh]) | 43,327 results |
| ((((((("Pregnant People"[Mesh]) OR ("Pregnant women")) OR ("Pregnancy"[Mesh])) OR ("Pregnant females")) OR ("Expecting mothers")) OR ("Expectant mothers")) AND ((((((((((Barriers) OR (Challenges)) OR (Obstacles)) OR (Hindrances)) OR (Limitations)) OR (Constraints)) OR (Difficulties)) OR (Facilitators)) OR (Enablers)) OR (Support))) AND ((((((((((((((("Oral healthcare utilization") OR ("Oral healthcare utilisation")) OR ("Oral care utilization")) OR ("Oral care utilisation")) OR ("Dental care utilization")) OR ("Dental care utilisation")) OR ("Dental service utilization")) OR ("Dental service utilisation")) OR ("Dental Care"[Mesh])) OR ("Use of dental services")) OR (" Dental care accessibility")) OR ("Access to dental care")) OR ("Dental visits")) OR ("Dental attendance")) OR ("Dental Health Services"[Mesh])) | 249 results |

**3b. Web of Science (Clarivate Analytics)**

Date of search: 08/04/2025

| "Pregnant People"(All Fields) | 1286 results |
| --- | --- |
| “Pregnant women"(All Fields) | 136,074 results |
| Pregnancy (All Fields) | 613,309 results |
| “Pregnant females” (All Fields) | 4,369 results |
| “Expecting mothers” (All Fields) | 183 results |
| “Expectant mothers” (All Fields) | 1,434 results |
| “Pregnant People” (All Fields)or “Pregnant women” or Pregnancy (All Fields) or “Pregnant females” (All Fields) or “Expecting mothers” (All Fields) or “Expectant mothers” (All Fields) | 661,942 results |
| Barriers (All Fields) | 1,026,374 results |
| Challenges (All Fields) | 3,464,211 results |
| Obstacles (All Fields) | 224,503 results |
| Hindrances (All Fields) | 38,315 results |
| Limitations (All Fields) | 1,140,325 results |
| Constraints (All Fields) | 985,796 results |
| Difficulties (All Fields) | 700,087 results |
| Facilitators (All Fields) | 63,510 results |
| Enablers (All Fields) | 29,688 results |
| Support (All Fields) | 9,777,453 results |
| Barriers (All Fields) or Challenges (All Fields) or Obstacles (All Fields) or Hindrances (All Fields) or Limitations (All Fields) or Constraints (All Fields) or Difficulties (All Fields) or Facilitators (All Fields) or Enablers (All Fields) or Support (All Fields) | 15,190,547 results |
| "Oral healthcare utilization" (All Fields) | 22 results |
| "Oral healthcare utilisation"(All Fields) | 6 results |
| "Oral care utilization"(All Fields) | 10 results |
| "Oral care utilisation"(All Fields) | 1 result |
| "Dental care utilization" (All Fields) | 376 results |
| "Dental care utilisation" (All Fields) | 39 results |
| "Dental service utilization" (All Fields) | 209 results |
| "Dental service utilisation" (All Fields) | 54 results |
| "Dental Care" (All Fields) | 13,509 results |
| "Use of dental services" (All Fields) | 496 results |
| “Access to dental care" (All Fields) | 894 results |
| " Dental care accessibility" (All Fields) | 10 results |
| "Dental visits" (All Fields) | 2,063 results |
| "Dental attendance" (All Fields) | 651 results |
| "Dental Health Services" (All Fields) | 792 results |
| "Oral healthcare utilization" (All Fields) or "Oral healthcare utilisation"(All Fields) or "Oral care utilization"(All Fields) or "Oral care utilisation"(All Fields) or "Dental care utilization" (All Fields) or "Dental care utilisation" (All Fields) or "Dental service utilization" (All Fields) or "Dental service utilisation" (All Fields) or "Dental Care" (All Fields) or "Use of dental services" (All Fields) or “Access to dental care" (All Fields) or " Dental care accessibility" (All Fields) or "Dental visits" (All Fields) or "Dental attendance" (All Fields) or "Dental Health Services" (All Fields) | 15,908 results |
| “Pregnant People” (All Fields)or “Pregnant women” or Pregnancy (All Fields) or “Pregnant females” (All Fields) or “Expecting mothers” (All Fields) or “Expectant mothers” (All Fields) AND Barriers (All Fields) or Challenges (All Fields) or Obstacles (All Fields) or Hindrances (All Fields) or Limitations (All Fields) or Constraints (All Fields) or Difficulties (All Fields) or Facilitators (All Fields) or Enablers (All Fields) or Support (All Fields) AND "Oral healthcare utilization" (All Fields) or "Oral healthcare utilisation"(All Fields) or "Oral care utilization"(All Fields) or "Oral care utilisation"(All Fields) or "Dental care utilization" (All Fields) or "Dental care utilisation" (All Fields) or "Dental service utilization" (All Fields) or "Dental service utilisation" (All Fields) or "Dental Care" (All Fields) or "Use of dental services" (All Fields) or “Access to dental care" (All Fields) or " Dental care accessibility" (All Fields) or "Dental visits" (All Fields) or "Dental attendance" (All Fields) or "Dental Health Services" (All Fields | 180 results |

**3c.** **ProQuest search**

Date of search: 08/04/2025

| Abstract ("Pregnant People") | 213 results |
| --- | --- |
| Abstract ("Pregnant women") | 24,849 results |
| Abstract (Pregnancy) | 63,953 results |
| Abstract (“Pregnant females”) | 597 results |
| Abstract (“Expecting mothers”) | 53 results |
| Abstract (“Expectant mothers”) | 348 results |
| Abstract ("Pregnant People") OR Abstract ("Pregnant women") OR Abstract (Pregnancy) OR Abstract (“Pregnant females”) OR “Expecting mothers” OR “Expectant mothers” | 73,560 results |
| Barriers | 148,909 results |
| Challenges | 513,516 results |
| Obstacles | 47,053 results |
| Hindrances | 3,851 results |
| Limitations | 199,770 results |
| Constraints | 218,487 results |
| Difficulties | 129,204 results |
| Facilitators | 14, 787 results |
| Enablers | 3949 results |
| Support | 571,785 results |
| Barriers OR Challenges OR Obstacles OR Hindrances OR Limitations OR Constraints OR Difficulties OR Facilitators OR Enablers OR Support | 1,598,127 results |
| "Oral healthcare utilization" | 6 results |
| "Oral healthcare utilisation" | 4 results |
| "Oral care utilization" | 2 results |
| "Oral care utilisation" | 0 results |
| "Dental care utilization" | 72 results |
| "Dental care utilisation" | 5 results |
| "Dental service utilization" | 44 results |
| "Dental service utilisation" | 14 results |
| "Dental Care" | 2211 results |
| "Use of dental services" | 82 results |
| “Access to dental care" | 186 results |
| " Dental care accessibility" | 3 results |
| "Dental visits" | 445 results |
| "Dental attendance" | 95 results |
| "Dental Health Services" | 50 results |
| "Oral healthcare utilization" OR "Oral healthcare utilisation" OR "Oral care utilization" OR "Oral care utilisation" OR "Dental care utilization" OR "Dental care utilisation" OR "Dental Care" OR "Use of dental services" OR “Access to dental care" OR " Dental care accessibility" OR "Dental visits" OR “Dental attendance" OR "Dental Health Services" | 2718 results |
|  | 28 results |

**3d. Open Access Theses and Dissertations**

Date of search: 08/04/2025

| "Pregnant People" | 94 |
| --- | --- |
| "Pregnant women" | 9333 |
| Pregnancy | 31,115 |
| “Pregnant females” | 361 |
| “Expecting mothers” | 35 |
| “Expectant mothers” | 216 |
| ("Pregnant People") OR "Pregnant women" OR (Pregnancy) OR “Pregnant females” OR “Expecting mothers” OR “Expectant mothers” | 34,853 |
| Barriers | 77,897 |
| Challenges | 257,585 |
| Obstacles | 33,838 |
| Hindrances | 1394 |
| Limitations | 132225 |
| Constraints | 87892 |
| Difficulties | 94736 |
| Facilitators | 9396 |
| Enablers | 3527 |
| Support | 454010 |
| (Barriers) OR each in bracket | 967,687 |
| "Oral healthcare utilization" | 2 |
| "Oral healthcare utilisation" | 1 |
| "Oral care utilization" | 0 |
| "Oral care utilisation" | 0 |
| "Dental care utilization" | 48 |
| "Dental care utilisation" | 6 |
| "Dental service utilization" | 29 |
| "Dental service utilisation" | 6 |
| "Dental Care" | 1694 |
| "Use of dental services" | 90 |
| “Access to dental care" | 137 |
| " Dental care accessibility" | 1 |
| "Dental visits" | 159 |
| "Dental attendance" | 64 |
| "Dental Health Services" | 73 |
| No bracket | 1943 |
|  | 0 |

**3e. CINAHL (EBSCO)**

Date of search: 09/04/2025

| "Pregnant People"(TX All Text) | 1,114 results |
| --- | --- |
| "Pregnant women" (TX All Text) | 85,057 |
| Pregnancy | 374,250 |
| “Pregnant females” | 1,066 |
| “Expecting mothers” | 297 |
| “Expectant mothers” | 19,073 |
| "Pregnant People"  OR "Pregnant women" OR | 397,050 |
| Barriers | 327,279 |
| Challenges | 643,612 |
| Obstacles | 71, 935 |
| Hindrances | 7,049 |
| Limitations | 510,537 |
| Constraints | 95,158 |
| Difficulties | 359,358 |
| Facilitators | 69,877 |
| Enablers | 11,163 |
| Support | 1,312,194 |
| OR | 1,984,765 |
| "Oral healthcare utilization" | 13 |
| "Oral healthcare utilisation" | 5 |
| "Oral care utilization" | 6 |
| "Oral care utilisation" | 0 |
| "Dental care utilization" | 696 |
| "Dental care utilisation" | 25 |
| "Dental service utilization" | 99 |
| "Dental service utilisation" | 27 |
| "Dental Care" | 24,075 |
| "Use of dental services" | 218 |
| “Access to dental care" | 520 |
| " Dental care accessibility" | 5 |
| "Dental visits" | 964 |
| "Dental attendance" | 263 |
| "Dental Health Services" | 1898 |
| OR | 25,829 |
| AND | 478 results |

**3f. PsycINFO (EBSCOHost)**

Date of search: 09/04/2025

| "Pregnant People"(TX All Text) | 230 |
| --- | --- |
| "Pregnant women" (TX All Text) | 12,664 |
| Pregnancy | 74,201 |
| “Pregnant females” | 986 |
| “Expecting mothers” | 50 |
| “Expectant mothers” | 1534 |
| OR | 75,994 |
| Barriers | 112,095 |
| Challenges | 277,189 |
| Obstacles | 24,961 |
| Hindrances | 2463 |
| Limitations | 154,924 |
| Constraints | 46,118 |
| Difficulties | 206,622 |
| Facilitators | 20,444 |
| Enablers | 3118 |
| Support | 717,017 |
| OR | 1,323,591 |
| "Oral healthcare utilization" | 2 |
| "Oral healthcare utilisation" | 795 |
| "Oral care utilization" | 1 |
| "Oral care utilisation" | 2312 |
| "Dental care utilization" | 50 |
| "Dental care utilisation" | 2 |
| "Dental service utilization" | 23 |
| "Dental service utilisation" | 1 |
| "Dental Care" | 2203 |
| "Use of dental services" | 45 |
| “Access to dental care" | 88 |
| " Dental care accessibility" | 4,066 |
| "Dental visits" | 213 |
| "Dental attendance" | 43 |
| "Dental Health Services" | 135 |
| OR | 2,389 |
| AND | 22 |

**3g. Scopus**

Date of search: 15/04/2025

| ABS ("Pregnant People") | 1261 documents |
| --- | --- |
| ABS (“Pregnant women") | 158,527 documents |
| ABS(Pregnancy) | 549,762 documents |
| ABS(“Pregnant females”) | 8227documents |
| ABS(“Expecting mothers”) | 249 documents |
| ABS(“Expectant mothers”) | 2146 documents |
| (ABS("Pregnant People")) OR (ABS(“Pregnant women"))OR (ABS(Pregnancy) )OR (ABS(“Pregnant females”) )OR (ABS(“Expecting mothers”))OR (ABS(“Expectant mothers”)) | 616,957 documents |
| ABS(Barriers) | 1,033,389 documents |
| ABS(Challenges) | 3,076,174 documents |
| ABS(Obstacles) | 296,727 documents |
| ABS(Hindrances) | 45,882 documents |
| ABS(Limitations) | 1,407,978 documents |
| ABS(Constraints) | 1,132,724 documents |
| ABS(Difficulties) | 988,592 documents |
| ABS(Facilitators) | 62,990 documents |
| ABS(Enablers) | 35,319 documents |
| ABS(Support) | 4,123,667documents |
| (ABS(Barriers)) OR (ABS(Challenges)) OR (ABS(Obstacles)) OR (ABS(Hindrances)) OR (ABS(Limitations)) OR (ABS(Constraints)) OR (ABS(Difficulties)) OR (ABS(Facilitators)) OR (ABS(Enablers)) OR (ABS(Support)) | 10,815,371 documents |
| ABS("Oral healthcare utilization") | 23 documents |
| ABS("Oral care utilization") | 8 documents |
| ABS("Dental care utilization") | 347 documents |
| ABS("Dental service utilization") | 302 documents |
| ABS("Dental Care") | 14,467 documents |
| ABS("Use of dental services") | 562 documents |
| ABS(“Access to dental care") | 950 documents |
| ABS (" Dental care accessibility") | 14 documents |
| ABS("Dental visits") | 3660 documents |
| ABS("Dental attendance") | 672 documents |
| ABS("Dental Health Services") | 541 documents |
| (ABS("Oral healthcare utilization")) OR (ABS("Oral healthcare utilisation")) OR (ABS("Oral care utilization")) OR (ABS("Oral care utilisation”)) OR (ABS("Dental care utilization")) OR (ABS("Dental care utilisation")) OR (ABS("Dental service utilization") ) OR (ABS("Dental service utilisation”)) OR (ABS("Dental Care")) OR (ABS("Use of dental services")) OR (ABS(“Access to dental care")) OR (ABS(" Dental care accessibility")) OR (ABS("Dental visits")) OR ( ABS("Dental attendance")) OR (ABS("Dental Health Services")) | 18,331 documents |
| ((ABS("Pregnant People")) OR (ABS(“Pregnant women"))OR (ABS(Pregnancy) )OR (ABS(“Pregnant females”) )OR (ABS(“Expecting mothers”))OR (ABS(“Expectant mothers”)) )AND ((ABS(Barriers)) OR (ABS(Challenges)) OR (ABS(Obstacles)) OR (ABS(Hindrances)) OR (ABS(Limitations)) OR (ABS(Constraints)) OR (ABS(Difficulties)) OR (ABS(Facilitators)) OR (ABS(Enablers)) OR (ABS(Support))) AND ((ABS("Oral healthcare utilization")) OR (ABS("Oral healthcare utilisation")) OR (ABS("Oral care utilization")) OR (ABS("Oral care utilisation”)) OR (ABS("Dental care utilization")) OR (ABS("Dental care utilisation")) OR (ABS("Dental service utilization") ) OR (ABS("Dental service utilisation”)) OR (ABS("Dental Care")) OR (ABS("Use of dental services")) OR (ABS(“Access to dental care")) OR (ABS(" Dental care accessibility")) OR (ABS("Dental visits")) OR ( ABS("Dental attendance")) OR (ABS("Dental Health Services"))) | 134 documents |

**3h**.  **Dentistry & Oral Sciences Source**

Date of search: 17/04/2025

| AB "Pregnant People" | 6 |
| --- | --- |
| AB "Pregnant women" | 991 |
| AB Pregnancy | 2048 |
| AB “Pregnant females” | 44 |
| AB “Expecting mothers” | 7 |
| AB “Expectant mothers” | 48 |
| AB( "Pregnant People" OR "Pregnant women" OR Pregnancy OR “Pregnant females” OR “Expecting mothers” OR “Expectant mothers”) | 2361 results |
| AB Barriers | 4,993 |
| AB Challenges | 11,750 |
| AB Obstacles | 535 |
| AB Hindrances | 88 |
| AB Limitations | 9,734 |
| AB Constraints | 557 |
| AB Difficulties | 6,141 |
| AB Facilitators | 259 |
| AB Enablers | 78 |
| AB Support | 15,994 |
| AB Barriers OR Challenges OR Obstacles OR Hindrances OR Limitations OR Constraints OR Difficulties OR Facilitators OR Enablers OR Support | 45,603 |
| AB "Oral healthcare utilization" | 8 |
| AB "Oral healthcare utilisation" | 1 |
| AB "Oral care utilization" | 565 |
| AB "Oral care utilisation" | 0 |
| AB "Dental care utilization" | 806 |
| AB "Dental care utilisation" | 806 |
| AB "Dental service utilization" | 105 |
| AB "Dental service utilisation" | 31 |
| AB "Dental Care" | 9,775 |
| AB "Use of dental services" | 260 |
| AB “Access to dental care" | 689 |
| AB " Dental care accessibility" | 10 |
| AB "Dental visits" | 1,154 |
| AB "Dental attendance" | 428 |
| AB "Dental Health Services" | 153 |
| AB"Oral healthcare utilization" OR ("Oral healthcare utilisation") OR ("Oral care utilization") OR ("Dental care utilization") OR ("Dental care utilisation") OR ("Dental service utilization") OR ("Dental care utilisation") OR ("Dental service utilization") OR ("Dental service utilisation") OR ("Dental Care") OR ("Use of dental services" OR “Access to dental care" OR " Dental care accessibility" OR " Dental care accessibility" OR "Dental visits" OR "Dental attendance"OR "Dental Health Services" | 11,494 |
| AB ((AB ((AB "Pregnant People") OR (AB "Pregnant women") OR (AB Pregnancy) OR (AB Pregnancy) OR (AB "Pregnant females") OR (AB “Expecting mothers”) OR (AB “Expectant mothers”))) AND (AB sq:652622bd-e202-4d36-8c18-04f0b1fce1a6 OR sq:079d1b00-701b-4ad9-8922 | 92 results |

**3i. Google scholar**

Date of search:16/04/2025

| Barriers and facilitators of oral healthcare utilization among pregnant women | 10 pages = 98 articles |
| --- | --- |

**1j.OAIster**

Date of search:16/04/2025

| kw:(("Pregnant women") OR (Pregnancy) OR ("Pregnant females”) )AND ((barriers)OR (enablers) OR (Facilitators)) AND (("Oral healthcare utilization") OR (Dental care utilization) OR ("Dental Care") OR (“Access to dental care") OR ("Dental visits") OR ("Dental Health Services")) | 29 results |
| --- | --- |
